# The consumption of experiential gifts is construed as more autonomy supportive and leads to greater gratitude, especially when they are given out of love

**DOI:** 10.3389/fpsyg.2023.1254789

**Published:** 2024-01-12

**Authors:** Rogelio Puente-Díaz, Judith Cavazos-Arroyo

**Affiliations:** ^1^Department of Business and Economics, Universidad Anáhuac México, Mexico City, Mexico; ^2^Centro Interdisciplinario de Posgrados, Universidad Popular Autónoma del Estado de Puebla, Puebla, Mexico

**Keywords:** consumption, experiences, gratitude, gifts, autonomy support

## Abstract

The purpose of this investigation was to examine the indirect influence of recalling the consumption of types of gifts, experiential and material, on gratitude by increasing autonomy support. In addition, we tested the conditional influence of the presumed motives of gift-givers from the perspective of gift recipients based on the postulates of Self-Determination Theory. First, participants were randomly to assigned to one of the following conditions: Consumption-of-experiential gift or consumption-of-material gift conditions. After, participants filled out a battery of questionnaires assessing autonomy support and gratitude. Results showed that the consumption of experiential gifts was construed as more autonomy supportive than the consumption of their material counterparts, which then had a positive relationship with gratitude. In experiment two, we tested the proposed mediator, autonomy support, by asking participants to either recall the consumption of the gift that was consistent with their true values or the consumption of an ordinary gift and completed a set of questions assessing autonomy support and gratitude. Results showed that recalling a gift consistent with consumers’ true values led to higher levels of autonomy support than recalling an ordinary gift, which was then positively correlated with gratitude. In the third experiment, we conducted a conceptual replication of experiment one and added the examination of the presumed motives of gift-givers as a potential moderator. Results replicated the significant mediation effect found in study 1 and showed that the conditional indirect effect was stronger when gift recipients attributed integrated motives to the gift-givers. The findings were discussed.

## 1 Introduction

Individuals play different roles in their lives. One of these roles is being a consumer. As consumers, individuals make thousands of consumption decisions. Recent theoretical developments on consumption posit different conceptualizations of the act of consumption. These conceptualizations have direct implications for how consumers make sense of these acts retrospectively and what these acts mean for consumers (Wherry and Woodward, 2019). Specifically, material culture emphasizes the role of meaning, meaning-making that comes from the act of consuming stuff (Miller, 2010). Stuff is a loosely conceptualized construct including tangibles and intangibles: Material products and experiences. Material culture is interested in the whole process of consumption, which goes beyond the act of purchasing something. As suggested by material scholars, “Consumption was not just buying things, it was the way we subsequently transformed the goods we had purchased (Miller, 2012, pp. 64).” Based on this conceptualization of consumption, we suggest that the transformation is not limited to goods, but also experiences and purchases made by others in the form of gifts, including tangibles and intangibles. In addition, we suggest that the transformation involves forming special memories and assessing when recollecting consumption episodes, what consumption episodes mean in terms of the satisfaction of the need for relatedness, competence, and autonomy (See Gilal et al., 2019 and Gul et al., 2021 for recent conceptualizations in marketing and Ryan and Deci, 2018 for recent conceptualizations in psychology).

Conceptual work on taste, a second approach to examining consumption, emphasizes the signaling function of the consumption of material objects and experiences, suggesting that consumption is a representation of one’s identity, the self (Bourdieu, 1984; Belk, 1988). This proposition is supported by additional conceptual developments suggesting that the self helps interpret, construe, and make sense of life events including consumption episodes (Oyserman, 2009). Individuals interpret what consumption episodes mean for the self. By combining these two approaches of consumption with recent developments in Self-Determination Theory and the consumption of experiences and material objects, we suggest that consumers extract, judge, and evaluate their consumption episodes in terms of how well they satisfy basic psychological needs (Ryan and Deci, 2018; Weingarten and Goodman, 2021). What this implies is that some consumption episodes might be perceived as more consistent with one’s true values than other types of consumption episodes with important affective and behavioral consequences. We consume material objects and experiences that we purchase and also that are purchased by others in the form of gifts. In this particular study, we focus on the consumption of gifts suggesting that reconstruing the consumption of experiential gifts versus material gifts should lead participants to feel greater autonomy support and this enhanced autonomy support should be positively related to greater feelings of gratitude. This is consistent with anthropological work suggesting that gifts could function as vehicles of identity for the self and others for both givers and recipients (Sherry, 1983). This proposed mechanism of greater autonomy coming from the consumption of experiential gifts would be qualified by the presumed motives behind the givers’ motivation as judged by the gift recipients. Scant attention has been paid to how the consumption of experiential and material gifts influences the construal of consumption episodes in terms of how consistent they are with consumers’ true values and their affective consequences (See Givi et al., 2023 and Gupta et al., 2023 for two recent literature reviews). Similarly, limited attention has been paid to how presumed motives, from the perspective of the gift recipient, might be a boundary condition for the positive influence of experiential gifts.

Hence, the purpose of this investigation is two-fold. First, we examine the influence of the consumption of types of gifts, experiential versus material, on autonomy support and the relationship between autonomy support and gratitude. Second, we test whether the proposed mediation model is qualified by the degree of self-determination of the presumed motives behind the givers’ motivation as judged by gift recipients. We first explain the basic principles of Self-Determination Theory (SDT) (Ryan and Deci, 2018).

### 1.1 Self-determination theory

Self-Determination Theory (SDT) is a motivational theory developed in social psychology with important applications for consumer psychology and consumer behavior (Ryan and Deci, 2018; Gilal et al., 2019). Within this motivational theory, organismic integration theory and basic psychological need theory are particularly relevant to understanding the dynamics of gift-giving from a motivational and affective approximation. Basic psychological need theory (Ryan and Deci, 2018) suggests the satisfaction of the need for autonomy, competence, and relatedness are key ingredients for consumers to show motivated behavior. The need for autonomy, relatedness, and competence represent more abstract, distal needs than the proximal needs of buying clothes to protect from the winter and taking a vacation to get a mental break. The assessment of the satisfaction of distal needs is likely to occur when consumers retrospect and infer meaning from past consumption episodes (Brockmeier, 2015). Regarding the role of autonomy, the focus of our investigation, this theoretical development suggests that the self is capable of transcending current experiences to make sense of what objects and experiences represent to the self (Ryan and Deci, 2018). One of the criteria used is how autonomy-supportive a given act is. We suggest that the satisfaction of the need for autonomy could come from evaluating in retrospect consumption episodes (Brockmeier, 2015). Specifically, we posit that some consumption episodes are evaluated as more consistent with consumers’ true values and self than others. The satisfaction of the need for autonomy should then have affective consequences.

Organismic integration theory (Ryan and Deci, 2018) focuses on the process of internalizing behaviors that could be at first extrinsically motivated. Gift-giving is social in nature and most consumers learn at a young age the dynamics of gift exchange (Wherry and Woodward, 2019). Gift giving from the recipients’ perspective could be evaluated as an act varying in their degree of self-determination from extrinsic (to look good socially), introjection (not to feel guilty), and identified (it is important to me to give and receive gifts, reciprocate) to integrated (for the love for this person) for buying and giving gifts. This evaluation occurs during the post-exchange stage of gift giving and receiving when gift recipients evaluate and re-evaluate the meaning of the gift and the presumed motives of the giver (Branco-Illodo et al., 2020).

In sum, the conceptual developments of basic psychological need theory and organismic integration theory are well suited to explain three important aspects of gifts that we seek to test empirically: (1) how different gifts are construed as more or less consistent with consumers’ true values (autonomy-supportive); (2) the affective consequences of an autonomy-supportive construal; and (3) how this whole process might be qualified by the presumed motives for giving gifts from the perspective of the recipient. Now we turn our attention to the examination of what types of gifts might be more satisfying of the need for autonomy and their affective consequences.

### 1.2 Types of purchases, gifts, and their affective outcomes

Consumer scholars have examined how different types of purchases and gifts, experiential versus material, lead to distinct affective outcomes (see Carter and Gilovich, 2012 for an example). Empirical evidence shows a happiness advantage of experiential purchases over material purchases (Weingarten and Goodman, 2021). A recent theoretical model posits that this experiential advantage could be mediated by need satisfaction (Weingarten and Goodman, 2021). Specifically, the model suggests that the satisfaction of the needs for relatedness, autonomy, and competence could be responsible for the affective advantage of experiential purchases. To our knowledge, this proposition has not been tested in the context of the consumption of gifts. According to self-determination theory (STD) (Ryan and Deci, 2018), individuals make sense of social situations by judging how well they satisfy their basic needs. We suggest that experiential and material gifts are consumption situations in which consumers construe some episodes as more consistent with the true values than others, leading to greater levels of autonomy support. Evidence from this proposition comes from a recent qualitative study on the best gift in which participants’ verbatims reflected the importance of receiving gifts consistent with their true selves (Branco-Illodo and Heath, 2020). Similarly, conceptual work on matches and mismatches between gift givers and recipients suggests that recipients focus on the long-term meaning of the gift, paying more attention to the meaning of the gift across time and less attention to the actual gift exchange. The consumption of gifts across time is often evaluated in terms of how consistent they are with recipients’ true values (Galak et al., 2016). From this, we posit and test the following hypothesis:

H1: Remembering the consumption of experiential gifts would be construed as more autonomy-supportive than remembering the consumption of material gifts.

If hypothesis 1 is correct, we suggest that this enhanced autonomy support should facilitate the experience of positive affective outcomes, besides happiness (Weingarten and Goodman, 2021).

### 1.3 Affective outcomes: the role of gratitude

Contrary to popular belief, the process of gift exchange does not always lead to positive affective outcomes for gift-givers or recipients. Gift-givers and recipients often experience anxiety, stress, and ingratitude, from the act of buying and receiving gifts (Sherry et al., 1993). Hence, it is important to examine affective outcomes in the form of gratitude as a function of need satisfaction and from the perspective of the recipients.

Gratitude is a social and moral emotion (Emmons and McCullough, 2004). The core mechanism of gratitude has two important components: (1) consumers see themselves as the receiver of a positive outcome and (2) the positive outcome comes from another person, from the efforts of others (Emmons and McCullough, 2004). Gift exchange represents a situation in which gift recipients feel grateful for the gift giver (Ward and Chan, 2015). Yet, we posit that if the consumption of experiential gifts is interpreted as more consistent with true values than the consumption of their material counterparts, then the recall of experiential gifts should lead to greater gratitude, by increasing the satisfaction of the need for autonomy. One previous study showed that experiential gifts led to greater gratitude because they represented more significant memories (Puente-Díaz and Cavazos-Arroyo, 2022). This study extends this line of research by making the claim the satisfaction of the need for autonomy is responsible for the gratitude advantage of experiential gifts. We posit and test the following hypothesis:

H2: Remembering the consumption of experiential gifts would have an indirect, positive relationship with gratitude by increasing the satisfaction of the need for autonomy.

The amount of gratitude experienced by gift recipients could be qualified by the presumed motives behind gift-giving from the perspective of the recipient. Some gifts might be construed as duties and obligations whereas others might be construed as true signs of care and love.

### 1.4 Boundary conditions: the degree of self-determination for giving gifts

**Figure 1:**
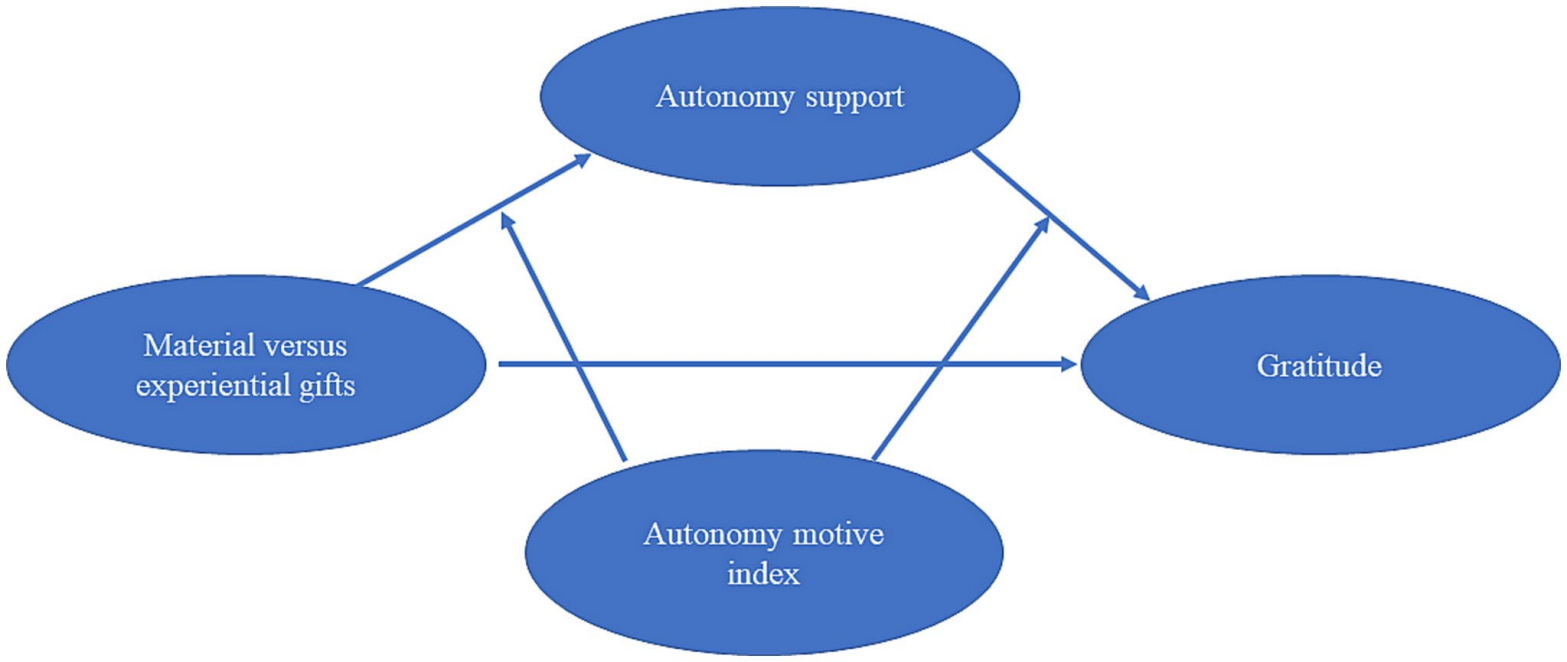
Conceptual model.

As suggested by consumer scholars, gift buying, giving, and consumption are popular activities around the world (Ward and Chan, 2015; Givi et al., 2023). Gift givers and recipients usually have shared and independent goals when buying, giving, and consuming gifts. The presumed motives for giving gifts could be a boundary condition limiting or enhancing how grateful consumers feel toward gift-givers. Motives for gift-giving could lie on a continuum from extrinsic, introject, identified, to integrated motives with important implications for the experience of gratitude. Integrated and identified motives are conceptualized as more self-determined than extrinsic and introjected motives (Ryan and Deci, 2018). We posit and test the following hypothesis:

H3: The positive indirect influence of experiential gifts on gratitude would be stronger if recipients infer higher integrated motives (autonomy index) for gift giving than if recipients infer lower integrated motives (see Figure 1 for conceptual model).

In sum, the purpose of the present investigation is twofold. First, we examine the direct influence of types of gifts on autonomy support and the indirect relationship with gratitude. Second, we test whether the proposed mediation model is qualified by the degree of self-determination of the presumed motives behind the givers’ motivation as judged by gift recipients. To accomplish this, we developed a questionnaire based on the principles of Organismic Integration Theory to assess presumed motives for gift-giving and buying.

## 2 Overview of studies

We conducted three studies to test our hypotheses. All studies received the approval of the human subjects committee of our university. In study 1, we manipulated type of gift and assessed its influence on autonomy support and gratitude. In study 2, we manipulated the proposed mediator, autonomy support, and assessed its influence of autonomy support and gratitude. Study 2 was conducted to overcome the limitation of measuring both the mediator and the outcome as in study 1. Study 3 was a conceptual replication of study 1 with the addition of the assessment of the presumed motives for gift-giving from the perspective of the recipient. We conducted a power analysis (G*Power, Faul et al., 2007) with the following specifications to determine the sample size needed: One independent variable with two levels, and power of 0.95 with an effect size of.26. Results recommended a sample of 196 participants. Hence, the sample size of 196 individuals was our goal in all three experiments.

## 3 Method of study 1

### 3.1 Participants

251 college students enrolled in two private universities in Mexico participated in study 1 (98% between the ages of 18–25, 59% females). Participants agreed to participate voluntarily without any form of compensation.

### 3.2 Procedure

Participants were randomly assigned to either the material or experiential gift consumption condition. The experimental instructions were taken from previous work on type of purchases and gifts (Puente-Díaz and Cavazos-Arroyo, 2022) and asked participants to focus on gift consumption as opposed to gift exchange:

Experiential condition: “For this activity, we would like you to think about an experiential gift that you have received with a price of around 1,000 pesos (50 US dollars). An experiential gift implies that a person spent money with the primary intention of you to have an experience- an event or a series of events that you have had and lived. We would like you to please focus on the consumption of this gift and not on the gift exchange. Please describe in the space provided below said gift consumption” (Puente-Díaz and Cavazos-Arroyo, 2022, pp. 3).

Material condition: “For this activity, we would like you to think about a material gift that you have received with a price of around 1,000 pesos (50 US dollars). A material gift implies that a person spent money with the primary intention of you to have a material possession- a material product that you obtain and keep in your possession. We would like you to please focus on the consumption of this gift and not on the gift exchange. Please describe in the space provided below said gift consumption (Puente-Díaz and Cavazos-Arroyo, 2022, pp. 3).”

### 3.3 Measures

Autonomy support and gratitude. All items used the prompt: remembering the consumption of this experiential (material) gift makes me feel? On a scale from “I do not feel like that at all” (1) to “I completely feel like that” (10). The four items for autonomy support were taken from the Basic Psychological Need Satisfaction and Frustration Scale (BPNSFS; Chen et al., 2015). The scores showed acceptable levels of internal consistency (α = 0.88). The items for gratitude were taken from the relevant literature (Weidman and Tracy, 2020) and were: I felt gratitude, I felt appreciative toward a specific person, I felt cared for, I felt fortunate, I felt like I had benefited from a specific person’s action, I felt lucky to know a specific person, I thought that a specific person who helped me should be acknowledged, and I wanted to express thanks. The scores showed acceptable levels of internal consistency (*α* = 0.86).

## 4 Results of study 1

We used the guidelines recommended by Hayes (2018) to test mediation models. First, results showed a significant effect of the experimental condition on autonomy support, *b* = 1.02, *p* < 0.001 (Bootstrap test, PROCESS model 4 as recommended by Hayes, 2018). While controlling for the experimental condition, the relationship between autonomy support and gratitude was also significant, *b* = 0.32, *p* < 0.001. Conversely, the influence of the experimental condition was not significant, *b* = 0.07, *p* = 0.63. Last, the indirect effect of the experimental condition on gratitude was significant, 0.32, CI = 0.19, 0.49 (see Table 1 for descriptive statistics). Hence, our results showed that remembering the consumption of an experiential gift led indirectly to greater gratitude by increasing autonomy support.

**Table 1:** Descriptive statistics for studies 1, 2, and 3.

	M	SD	M	SD
**Study 1**	Experiential		Material	
Autonomy support	8.89	1.18	7.87	1.85
Gratitude	9.41	1.03	9.01	1.27
**Study 2**	Autonomy supportive		Ordinary	
Autonomy support	8.91	1.42	7.76	2.09
Gratitude	9.26	1.31	8.61	1.96
**Study 3**	Experiential		Material	
Autonomy support	8.69	1.44	8.08	1.89
Gratitude	9.30	1.26	9.19	1.10
Integrated motives	8.64	1.32	8.65	1.09

### 4.1 Brief discussion

Our results showed that the consumption of experiential gifts led to greater autonomy support, providing initial support for hypothesis 1. In addition, experiential gifts had an indirect effect on gratitude, supporting hypothesis 2. This result was consistent with previous studies (Puente-Díaz and Cavazos-Arroyo, 2022). One limitation of study 1 is that we did not manipulate the proposed mediator, autonomy support. As suggested by methodology scholars, it is important to manipulate the mediator (Spencer et al., 2005), when testing mediation models. Hence, in study 2, participants were asked to bring to mind a gift that was either consistent with their values, autonomy supportive, or an ordinary gift, and assessed the influence of this manipulation on autonomy support and gratitude.

## 5 Method of study 2

### 5.1 Participants

As in study 1, participants were 181 college students enrolled in two private universities in Mexico (88% between the ages of 18–25, 65% females). Participants agreed to participate voluntarily without any form of compensation. We were not able to achieve the goal of having 196 participants due to mandatory lockdowns from the COVID pandemic.

### 5.2 Procedure

Participants were randomly assigned to either the autonomy-supportive-gift condition or the ordinary-gift condition. We asked participants, as in study 1, to focus on the consumption of either type of gift. The instructions were:

Autonomy-supportive-gift condition: For this activity, we would like to think about a gift that had been consistent with your values and beliefs that you received with a price of around 500 pesos (25 US dollars). A gift consistent with your values and beliefs implies that the gift truly reflected who you are as a person. “We would like you to please focus on the consumption of this gift and not on the gift exchange. Please describe in the space provided below said gift consumption” (Puente-Díaz and Cavazos-Arroyo, 2022, pp. 3).

Ordinary-gift condition: For this activity, we would like you to think about a common ordinary that you received with a price of around 500 pesos (25 US dollars). A common gift implies that the gift achieved its objective, without being special. “We would like you to please focus on the consumption of this gift and not on the gift exchange. Please describe in the space provided below said gift consumption” (Puente-Díaz and Cavazos-Arroyo, 2022, pp. 3).

### 5.3 Measures

The same measures as study 1 were used to assess autonomy support and gratitude. Both scores showed acceptable levels of internal consistency (*α* = 0.93 and *α* = 0.93, respectively).

## 6 Results of study 2

We used the same guidelines as in study 1 to test the mediation model (Hayes, 2018). First, results showed a significant effect of the experimental condition on autonomy support, *b* = 1.15, *p* < 0.001 (PROCESS model 4, Hayes, 2018). While controlling for the experimental condition, the relationship between autonomy support and gratitude was also significant, *b* = 0.62, *p* < 0.001. The effect of the experimental condition on gratitude was not significant, *b* = −0.06, *p* = 0.70. Last, the indirect effect of the experimental condition on gratitude was significant, 0.71, CI = 0.38, 1.04 (see Table 1 for descriptive statistics). Hence, our results showed that recalling the consumption of a gift consistent with recipients’ true values led indirectly to greater gratitude by increasing autonomy support.

### 6.1 Brief discussion

Study 2 addressed one important limitation by manipulating the proposed mediator, autonomy-supportive gifts, and assessing its impact on gratitude. Results showed additional support for hypotheses 1 and 2. Participants who brought to mind a gift consistent with their values reported greater autonomy support, which then had a positive relationship with gratitude. In the next experiment, we wanted to extend the examination of type of gift by exploring one potential moderator of the relationship between type of gift and gratitude. This moderator was the presumed motives for giving and buying gifts of the gift-giver from the perspective of the gift recipient.

## 7 Method of study 3

### 7.1 Participants

As in studies 1 and 2, 265 college students enrolled in two private universities in Mexico participated (97% between the ages of 18–25, 67% females). Participants agreed to participate voluntarily without any form of compensation.

### 7.2 Procedure

Participants were randomly assigned to one condition: consumption of experiential versus consumption of material gift conditions.

### 7.3 Measures

The same measures as studies 1 and 2 were used to assess the satisfaction of the need for autonomy and gratitude. Both scores showed acceptable levels of internal consistency (α = 0.89 and α = 0.90, respectively). To assess presumed motives for gift-giving and buying, we consulted the literature on gift exchange and on Organismic Integration theory to write items seeking to assess: Extrinsic, Introjected, Identified, and Integrated motives. We wanted to have at least three items per dimension (See Appendix for list of items).

### 7.4 Confirmatory factor analysis for the gift motives scale

Given that we developed a new scale, we tested its measurement properties with a Confirmatory Factor Analysis. We first tested a measurement model with four latent variables and twelve observed variables. The latent variables were extrinsic, introjected, identified, and integrated with three items each. Results showed an acceptable model fit χ^2^ = 362.54, *p* < 0.001 (df = 59), RMSEA = 0.15, CFI = 0.93, and TLI = 0.91. All factor loadings were significant and in the expected direction (ranging from 0.64 to 0.94). The bivariate correlations between the latent variables showed a strong correlation between extrinsic and introjected motives (0.96). This strong correlation suggested that gift recipients were not able to distinguish between external and introjected reasons. Given these results, we test an additional measurement model with three latent variables and the same twelve observed variables, combining into a single factor extrinsic and introjected motives. Results for the measurement model showed a better model fit χ^2^ = 173.02, *p* < 0.001 (df = 49), RMSEA = 0.19, CFI = 0.97, and TLI = 0.96. All factor loadings were significant and in the expected direction (ranging from 0.52 to 0.94). The bivariate correlations between the latent variables were: 0.73 between extrinsic/introjected motives and identified motives, −0.71 between extrinsic/introjected motives and integrated motives, and − 0.13 between identified and integrated motives. The h coefficients had acceptable levels: extrinsic/introjected (0.95), identified (0.90), and integrated (0.81). Given these results and some recommendations from the literature (Van der Kaap-Deeder et al., 2020), we calculated an autonomy motive index by subtracting the scores of extrinsic/introjected motives from the sum of the identified and integrated motives.

## 8 Results of study 3

We utilized the same guidelines as in studies 1 and 2 to test our mediation model before assessing the contribution of the moderator (Hayes, 2018). First, there was (PROCESS, model 4, Hayes, 2018) a significant effect of the experimental condition on autonomy support, *b* = 0.70, *p* = 003. While controlling for the experimental condition, the relationship between autonomy support and gratitude was also significant, *b* = 0.39, *p* < 0.001. Conversely, the influence of the experimental condition was not significant, *b* = −0.11, *p* = 0.41. Last, the indirect effect of the experimental condition on gratitude was significant, 0.27, CI = 0.11, 0.43 (see Table 1 for descriptive statistics).

### 8.1 Moderated mediation

We used the same recommendations (Hayes, 2018) to test our moderated mediation model. Given that we wanted to focus on autonomy support, we used the autonomy motive index just described to test our moderated mediation model. A bootstrap test (PROCESS, model 58, Hayes, 2018) showed that, for the mediator autonomy support, there was not a significant interaction between type of gift and the autonomy motive index, *b* = 0.01, *p* = 0.52. A second equation showed a significant interaction between autonomy support and the autonomy motive index, *b* = −0.013, *p* < 0.001. The most important interpretation is that the indirect effect of the consumption of experiential gifts on gratitude, through its influence on autonomy support, was only significant at medium and levels of the autonomy motive index as inferred by gift recipients (see Table 2 for full results and Table 3 for the interpretation of indirect condition effect).

**Table 2:** Full results of moderated mediation.

Test of moderated mediation for the outcome variable of gratitude		
Variable	Coefficient	*p* value
Experimental condition	−0.02	0.87
Autonomy support	0.86	< 0.001
Autonomy motive index	0.13	< 0.001
Interaction between	−0.013	< 0.001
autonomy support and		
autonomy motive index		

**Table 3:** Conditional indirect effects of the consumption of experiential gifts on gratitude.

Indirect effect:			
Consumption of experiential gifts influences gratitude indirectly via autonomy support			
	Effect	Lower level-CI	Upper level-CI
16th percentile of integrated motives	0.21	−0.13	0.42
50th percentile of integrated motives	0.18	0.05	0.38
84th percentile of integrated motives	0.11	−0.10	0.47

*Conditional indirect effect on the values of autonomy motive index.

### 8.2 Brief discussion

Our results showed additional support for the indirect effect of the consumption of experiential gifts on gratitude by increasing autonomy support. More importantly, our results showed that this indirect effect was moderated by autonomous motives for gift giving and buying from the perspective of gift recipients. The indirect effect was only significant at medium levels of autonomous motives. These findings could constitute a relevant, small contribution to our understanding of gift exchange from the perspective of self-determination theory.

## 9 General discussion

Across three studies, our results showed that the consumption of experiential gifts was construed as more autonomy supportive than the consumption of material gifts. This experiential advantage led to higher gratitude, yet the indirect effect was qualified by how autonomous the presumed motives for gift-giving and buying were from the perspective of the gift recipient. Consequently, two hypotheses were supported and one partially supported. We discussed the potential theoretical and applied contributions.

### 9.1 Theoretical contributions

The satisfaction of consumers’ needs is at the heart of marketing (Tybout and Calder, 2010). Borrowing a term from evolutionary psychology, the satisfaction could involve proximal needs such as taking a vacation to reduce stress, or more distal needs such as taking a vacation to a place consistent with my love for archeology. Both types of needs, proximal and distal can be satisfied with the same consumption experience, taking a vacation. It is just that the satisfaction of more distal needs is more likely to occur during the process of making sense and extracting meaning from the consumption episode. This process is likely to focus on more abstract needs as well. Given that there could be many potential distal needs, one way to extract meaning more efficiently is to assess how the consumption episode satisfies the need for competence, relatedness, and autonomy, the latter one as the focus of our investigation, as originally suggested by Weingarten and Goodman (2021). Our results showed that the consumption of experiential gifts was construed as more autonomy-satisfying, providing support for hypothesis one. Our results were consistent with a recent model developed to explain the advantage of experiential purchases and extend the implications to the consumption of gifts (Weingarten and Goodman, 2021). Our results have implications for a recent review suggesting the underutilization of Self-Determination Theory and need satisfaction theory to explain relevant motivational outcomes in consumer behavior. In addition, based on a recent review of the literature on gift dynamics (Givi et al., 2023), our results also have implications for a parsimonious model of gift outcomes based on the satisfaction of the need for autonomy, relatedness, and competence.

As suggested by need satisfaction theory (Ryan and Deci, 2018), the satisfaction of the need for autonomy should be related to positive affective outcomes. Our findings were in line with this proposition. Specifically, we found an indirect influence of the consumption of experiential gifts on gratitude by satisfying the need for autonomy, supporting hypothesis two. We see two conceptual implications of our results. First, our findings showed additional evidence of the utility of Self-Determination Theory to explain important affective outcomes coming from consumption. Second, based on the literature review of gift dynamics (Givi et al., 2023), more attention should be paid to affective outcomes coming from buying, giving, and receiving gifts. As suggested by anthropological work on gifts (Sherry et al., 1993), gift exchange is full of mixed, complex, intense emotional experiences of anxiety, stress, and ingratitude, among others. Thus, our results made a small contribution to understanding an important affective outcome of receiving gifts in the form of gratitude.

Last, our results showed that the presumed motives for gift buying and giving from the perspective of recipients mattered. Yet contrary to our hypothesis, it was at medium levels of autonomy motives that consumers were more likely to experience gratitude from the consumption of experiential gifts. At low and high levels of autonomy motives, the indirect effect of type of gift on gratitude was not significant. To our knowledge, this was the first study to assess presumed motives from a self-determined perspective in which more autonomy motives were hypothesized to lead to more positive affective outcomes. Our results showed again the utility of Self-Determination Theory to understand consumer behavior and were in line with the proposition that during the consumption of gifts, consumers make sense of the episodes and extract meaning from them (Branco-Illodo et al., 2020). For this particular case, the act of consuming an experiential gift led to greater gratitude, but this was especially so when gift receipts inferred medium levels of autonomy reasons in the gift-giver process of decision-making.

### 9.2 Applied implications

Material culture emphasizes the role of meaning, meaning-making that comes from the act of consuming stuff (Miller, 2010). In addition, recent work on best gifts and failed gifts (Branco-Illodo et al., 2020; Branco-Illodo and Heath, 2020) shows that gift recipients engage in meaning extraction, evaluation, and reevaluation of the significance of gifts. It looks like consumers often or at least occasionally assess and reassess the significance of gifts received with important affective outcomes. Consequently, marketers should be well-advice to organize and facilitate post-gift assessment and reassessment by organizing activities on social media. For example, service providers such as restaurants and entertainment venues could encourage consumers to upload their stories about recent dining and concert experiences. This dynamic is likely to lead consumers to enjoy and re-enjoy telling their experiences and reading the experiences of other consumers. When telling their experiences and extracting meaning from them, consumers are likely to feel good about themselves (need for competence), connected to important others (need for relatedness), and feel that the experience was consistent with their true values (need for autonomy) with positive affective outcomes. These positive affective outcomes could facilitate important consumption behaviors such as positive word of mouth and repurchase intentions.

### 9.3 Limitations

Our research had the following limitations. First, our sample did not represent well the whole population of Mexico given that it was a sample of convenience. Second, we used a cross-sectional research design, which prevents us from understanding how gift dynamics, including the natural assessment of meaning and reevaluation of consumption experiences of gifts, unfold. We asked participants explicitly to bring to mind the consumption of gifts, yet we do not know if the same results would hold from the spontaneous retrieval of consumption episodes. Our third limitation is that we only focused on gratitude. For example, one study examined the implications of gift consumption for the experience of nostalgia (Puente-Díaz and Cavazos-Arroyo, 2021). Future research could explore additional affective outcomes.

In sum, we showed support for the idea that the consumption of experiential gifts was more autonomy supportive, which was then positively related to greater gratitude. This indirect effect was qualified by the degree of integrated motives for buying and giving gifts as inferred by gift recipients. We look forward to continuing to observe the increased attention paid to gift dynamics in consumer research.

## Data availability statement

The raw data supporting the conclusions of this article will be made available by the authors, without undue reservation.

## Ethics statement

The studies involving humans were approved by Universidad Anahuac Review Board. The studies were conducted in accordance with the local legislation and institutional requirements. The ethics committee/institutional review board waived the requirement of written informed consent for participation from the participants or the participants’ legal guardians/next of kin because it could not be obtained on the internet.

## Author contributions

RP-D: Conceptualization, Data curation, Validation, Writing – original draft, Writing – review & editing. JC-A: Conceptualization, Investigation, Supervision, Writing – review & editing.

## Appendix

For this section, we would like you to think about the person that gave you the gift that you just described.

Using the scale that goes from (1) not true at all to (10) completely true.

Why do you think this person decided to buy you a gift?

1. For commitment or obligation
2. Because I am important to this person
3. Not to feel guilty
4. To look good socially
5. Because it is part of the tradition
6. To make me feel happy
7. Not to look bad
8. Out of love for me
9. To preserve the relationship
10. Because giving gifts is consistent with her or his values
11. Not to feel bad about her or himself
12. Because giving gifts is consistent with her or his way of being

Scoring key:

Extrinsic: 1, 4, 5,

Introjected: 3, 7, 11.

Identified: 9, 10, 12.

Integrated: 2, 6, 8.
